# Rapid Cure of Whooping Cough

**Published:** 1888-10

**Authors:** 


### RAPID CURE OF WHOOPING COUGH.

The treatment of this disease, as published by one of our Norwegian brethren, is very interesting, in the fact that the remedy is said to have been of extraordinary efficacy in his hands, and that it seems to demonstrate the parasitic origin of the group of systoms to which are given the name whooping cough.

Says Dr. Mohn, my eldest child, about six years ago, then three years old, was attacked with scarlatina and whooping cough. The fits of coughing had commenced eight days after the eruption, which was light, the infant not being confined to bed more than two or three days; the whooping cough did not present much gravity. About five weeks from the debut of the fits, the room was disinfected by means of sulphur fumigations. Some hours before the disinfection there occurred a very violent fit of coughing, but afterwards there were none. A sister, a little older than this one, who had the premonitory symptoms of whooping cough, was immediately cured. Four years later, M. Mohn had one of his children, aged three years, attacked. Three brothers and sisters had been attacked in the meanwhile, but did not present any symptoms very serious. This little child had also a bronchitis, and epistaxis had much enfeebled her. M. Mohn expected an unfavorable issue ; all the internal medications had been tried ; inhalations of chloroform and phenic acid, but without avail. Recollecting then that fumigations of sulphurous acid had cured the disease in the eldest child, he applied the remedy in this case. In the following night there were but two slight paroxysms, and the next day the cough had disappeared. The other children were cured by the same applications.

M. Mohn cites other cases :

A little child of five months, with cough of five weeks duration, and a boy of four years, with cough for twelve days, were cured as by magic after one sulphurous fumigation. An infant of three years, with cough of five weeks duration, and three brothers and sisters, were cured instantaneously, after one fumigation.

The following is the method of operation :

In the bedroom and in the room usually occupied by the patients, are to be suspended the clothes, playthings and all articles incapable of being washed. 25 grammes of sulphur for every cubic metre, are to be burned in the rooms to be disinfected, and they are to be closed for five hours. Then the chamber is to be freely aired and the articles exposed to the air. Thus the patient will sleep in a room and in a bed completely disinfected, and the whooping cough is cured.

This mode of treatment is certainly very simple and easy to employ, and may be called upon to render service where all other modes of treatment have failed.—*Union Medicale du Canada.*

